# Medical Student Comfort With Procedural Skills Performance Based on Elective Experience and Career Interest

**DOI:** 10.7759/cureus.12374

**Published:** 2020-12-30

**Authors:** Bright Huo, Wyatt MacNevin, Michael Smyth, Stephen G Miller

**Affiliations:** 1 Faculty of Medicine, Dalhousie University, Halifax, CAN; 2 Anesthesia, Pain Management & Perioperative Medicine, Dalhousie University, Halifax, CAN; 3 Emergency Medicine, Dalhousie University, Halifax, CAN

**Keywords:** elective, comfort, medical student, exposure, procedural skills, clinical skills, medical education, career interest

## Abstract

Introduction

Despite increased efforts, studies suggest that exposure to procedural skills in undergraduate medical training is insufficient. As medical students have low self-reported competence in many skills, a significant concern is that medical students are underprepared for a clerkship. Furthermore, pre-clerkship electives selected based on student career interests can provide students with additional skills learning opportunities. The impact of career interest and elective choice on student comfort with procedural skills is unclear. This study examines the relationship between student procedural skills comfort, career interest, and elective choices.

Materials and methods

An evidence-based questionnaire was synthesized following a literature search using PubMed, Embase, and Google Scholar. Surveys were completed by second-year medical students. A Likert scale was used to evaluate students’ exposure, comfort, and motivation to learn common procedural skills. Descriptive, Pearson’s chi-square and Spearman’s rho correlation coefficient analyses were performed to evaluate the relationship between career interests, elective exposure, and procedural skills.

Results

Medical students (>60%) reported poor comfort levels for most skills, despite >80% of students displaying high motivation to learn. Elective choice impacted student comfort levels as students who completed electives in anesthesiology were more comfortable with performing intubation (23% vs 10%, p = 0.026) and IV insertion (38% vs 13%, p = 0.002). Those with surgical career interests were less comfortable performing Foley catheter insertion in males (7% vs 5%, p = 0.033) and in females (7% vs 5%, p = 0.008).

Conclusions

This study supports that medical students feel low levels of comfort with performing procedural skills despite high motivation for learning. Comfort was influenced by both career interest and elective experience. Programs aiming to increase students’ comfort levels in performing procedural skills should adapt curricula toward increasing early exposure to these skills.

## Introduction

Clinical skills training has traditionally been limited during the first two years of medical school and while current curricula incorporate early formal instruction in basic procedural skills, training can be inconsistent and advanced procedures receive less exposure [1]. Additionally, a discrepancy exists between perceptions of faculty and medical students on whether a sufficient amount of simulation-based teaching is present in undergraduate curricula, as medical students have substantially less procedural skills experience than what is expected by residency program directors, nurses, physicians, and other healthcare professionals in the workplace [2-6]. Despite increased efforts, studies suggest that exposure to procedural skills in undergraduate medical training is insufficient, as medical students have low self-reported competence in many skills [2,3,7].

Medical students entering clerkship have low confidence and high levels of anxiety in performing common procedural skills such as suturing, NG tube placement, and IV catheterization [4,8]. In graduating medical students, up to 73% have never performed intubation and 44% have never performed IV catheter insertion [9]. As senior medical students may not possess the expected competence in simple procedural skills, a significant concern is that medical students are underprepared for clerkship and subsequently, residency [3,7,10,11].

The relationship between procedural confidence and competence is complex, and some literature suggests that the two are mutually exclusive [12]. As confidence in procedural skills has been shown to improve with exposure, self-perceived confidence in procedural skill performance is commonly used as an assessment marker in medical education [13-15]. For instance, one study demonstrated that an additional four-hour course improved self-reported proficiency, confidence, and reduced anxiety in performing knot-tying, suturing, NG tube insertion, IV catheterization, and bladder catheterization [16]. Additional training in procedural skills has been shown to improve clerkship performance in students’ third year of medical school [8,17-19]. However, the best method of addressing deficits in skills confidence and which skills to target are still unclear from an undergraduate medical education perspective.

The proposed benefit of exposure asserts the need to explore the impact of elective experience and career interest on skills performance. Furthermore, the relationship between students’ motivation to learn common procedural skills and the perceived likelihood of future skills use has yet to be determined. This knowledge would inform an approach to improving procedural skills-related competence in medical undergraduate education. The objective of this study is to investigate the perceived levels of comfort, exposure, motivation, and the likelihood of future use, of common procedural skills in medical students prior to clerkship.

## Materials and methods

Surveys were distributed to second-year pre-clerkship medical students at Dalhousie University in Halifax, Nova Scotia, Canada via Opinio (Object Planet, Oslo, Norway), and data were collected anonymously. Survey respondents were applicants to the Pre-Clerkship Residency Exploration Program (PREP), an elective program that provides students with practical exposure to various specialties. The study period lasted two and a half weeks and participation in the study was voluntary with no exclusion criteria applied. Ethics approval was provided by the Nova Scotia Health Authority Research Ethics Board (File No. 1023087).

Survey design

To inform the development of our cross-sectional survey, a literature search was completed from December 2 to 21, 2019. The search was completed in PubMed, Embase, and Google Scholar, and the articles were screened by two rounds of reviews to capture articles assessing medical student confidence in procedural skills performance. The inclusion criteria included studies that examined the confidence of undergraduate medical students in performing procedural skills. Reviews, surveys, and qualitative studies were included. Non-English studies were excluded and those conducted in North America were preferred. Articles examining postgraduate medical trainees were excluded.

The first round of screening consisted of reviewing titles and abstracts, while full-text articles were reviewed during the second round of screening. Conflicts were resolved by a third, faculty team member. A total of 14 articles relevant to our research question were yielded. ClinicalTrials.gov was searched on December 22, 2019, to identify studies that were ongoing, unpublished, or withdrawn and yielded no additional articles.

Literature conflicts when describing precisely which procedural skills pre-clerkship medical students should be expected to attain competence in performing. Thus, both the principal investigator and co-investigator independently identified pertinent procedural skills for assessment, with consideration of the literature on undergraduate medical skills training for pre-clerkship education. Proficiency in skills expected at a postgraduate level such as IUD insertions and joint aspirations were included as they have been assessed in other studies. After a full review, all investigators collaboratively settled disagreements to generate a final list of procedural skills.

Data were collected on demographics such as age, gender, marital status, education, desired practice location, and rural/urban upbringing. Previous clinical electives and specialty interest were also assessed. Survey questions evaluated their level of exposure through academic and clinical experience, comfort, and motivation to learn procedural skills using a five-point Likert scale. The following skills were assessed: intramuscular (IM) injection, subcutaneous (SC) injection, intravenous (IV) insertion, intradermal (ID) insertion, intraosseous (IO) insertion, intubation, suturing, phlebotomy, nasogastric (NG) tube insertion, Foley catheter insertion (both male and female), intrauterine device (IUD) insertion, digital rectal examination (DRE), speculum exam, breast exam, bag-mask ventilation, superficial wound care, throat swabbing, lumbar puncture, central venous catheterization (CVC) and joint aspiration.

Statistical methods and data analysis

Data were exported into IBM Statistical Package for the Social Sciences (SPSS) software (version 25, IBM, New York, United States) and demographic characteristics were expressed as frequencies and percentages. Pearson’s chi-square tests and Spearman’s rho correlation coefficient analysis were used to analyze the relationship between demographic factors and procedural skills. Analyses were also performed between career interest, elective exposure, and skills. A 95% confidence interval was used with statistical significance set at p < 0.05.

## Results

Demographics

A total of 53/116 (45.7%) second-year medical students from Dalhousie University responded to the survey (Table 1). Fifty-one percent of students were between the age of 20 and 24 years and 87% of participants reported both an urban upbringing and a desire to work in an urban area.

**Table 1:** Demographic characteristics of PREP respondents (N = 53) PREP: Pre-Clerkship Residency Exploration Program

		Frequency	Percentage (%)
Gender	Male	21	40
	Female	32	60
Age	20-24	27	51
	25-28	23	43
	29+	3	6
Upbringing	Urban	46	87
	Rural	7	13
Desired Practice Location	Urban Community	46	87
	Rural Community	7	13
Desired Practice Setting	Community Clinic	13	25
	Hospital-based Clinic	21	40
	Academic Centre	17	32
	Other	2	4

The majority of students were interested in careers in medical specialties (47%) or expressed that they were undecided at the time of the survey (45%) (Table 2). Many students had experienced an elective in medicine (57%) as well as surgery (53%), while only 30% of students had experienced an elective in anesthesiology.

**Table 2:** Previous electives and clinical interests of PREP participants (N = 53) PREP: Pre-Clerkship Residency Exploration Program

		Frequency	Percentage (%)
Previous Electives	Medicine	30	57
	Surgery	28	53
	Anesthesia	16	30
Clinical Interests	Medicine	25	47
	Surgery	4	8
	Undecided	24	45

Comfort in procedural skills

Students reported poor comfort for most procedural skills, as ≥60% of the cohort felt uncomfortable performing bag-mask ventilation (60.0%), breast examinations (60.4%), phlebotomy (62.3%), NG tube insertions (64.1%), throat swabbing (66%), intubations (67.9%), and Foley catheter insertions in females (79.2%) and males (81.1%) (Table 3). Even for the top-performing skills, more than a third of students reported low comfort in performing IM injections (36%), suturing (38%), and SC injections (38%).

**Table 3:** Comfort and exposure to an evidence-based list of procedural skills IV: intravenous; IO: intraosseous; IM: intramuscular; SC: subcutaneous; NG: nasogastric; IUD: intrauterine device; DRE: digital rectal examination

	Comfort		Exposure	
Skill	Low/Very Low	High/Very High	Low/Very Low	High/Very High
Intubation	67.9%	9.4%	54.7%	26.4%
IV Insertion	50.9%	22.6%	45.3%	30.2%
Suturing	37.7%	28.3%	33.9%	39.6%
Phlebotomy	62.3%	9.4%	62.2%	13.2%
IO Insertion	73.6%	7.5%	71.2%	5.7%
IM Injections	35.8%	28.3%	22.6%	30.2%
SC Injections	37.7%	18.9%	37.7%	24.5%
Intradermal Injections	52.8%	15.1%	56.6%	13.2%
NG Tube Insertion	64.1%	11.3%	60.3%	7.5%
Foley Catheter (Male)	81.1%	5.7%	79.2%	1.9%
Foley Catheter (Female)	79.2%	5.7%	77.3%	1.9%
IUD Insertion	81.1%	1.9%	84.9%	9.4%
DRE	64.1%	9.4%	64.2%	9.4%
Speculum Examination	67.9%	5.7%	60.4%	9.4%
Breast Examination	60.4%	11.3%	62.3%	9.4%
Bag-Mask Ventilation	60.4%	18.9%	64.2%	28.3%
Lumbar Puncture	86.8%	1.9%	88.7%	1.9%
Central Venous Catheter	90.6%	0.0%	88.7%	1.9%
Joint Aspiration	92.4%	0.0%	94.3%	0.0%
Superficial Wound Care	77.4%	5.7%	75.5%	7.5%
Throat Swabbing	66.0%	5.7%	79.2%	11.3%

Previous electives influenced student comfort as those that completed electives in surgery felt less comfortable with NG insertion (7% vs. 13%, p = 0.05), Foley catheter insertion in males (0% vs 8%, p = 0.027), and Foley catheter insertion in females (8% vs 0%, p = 0.007) compared to those without surgical electives. Students interested in surgery reported less comfort with performing ID injections (7% vs 18%, p = 0.01), NG insertion (8% vs 21%, p = 0.041), Foley catheter insertion in males (5% vs 7%, p = 0.033), and females (5% vs 7%, p = 0.008), IUD insertion (0% vs 7%, p = 0.046), speculum examinations (0% vs 21%, p = 0.008), and breast examinations (13% vs 14%, p = 0.003). Contrarily, those interested in medicine reported increased comfort with performing ID injections (17% vs 14%, p = 0.047) and breast exams (17% vs 10%, p = 0.029). For students that completed electives in anesthesiology, there was increased comfort with intubation (23% vs 10%, p = 0.026) and IV insertion (38% vs 13%, p = 0.002) skills.

Skill exposure

Medical students reported low exposure to advanced skills such as IO insertion (71.2%), lumbar puncture (89%), CVC insertion (89%), and joint aspiration (94%) (Table 3). Most participants (≥60%) reported low exposure to basic skills including phlebotomy (62.2%), breast examination (62.3%), bag-mask ventilation (64.2%), DRE (64.2%), and throat swabbing (79.2%). Skills that had the highest amount of exposure included IM injections (30%), IV catheter insertion (30%), and suturing (40%).

Skills exposure was influenced by elective experience as students that completed surgical electives were more likely to gain exposure to DREs compared to those without surgical electives (21% vs 5%, p = 0.03). Furthermore, those who completed medicine-based electives had lower rates of exposure with performing NG tube insertion (3. 8% vs 11.1%, p = 0.041) and intubations (19% vs 37%, p = 0.036) when compared to students who did electives in non-medical fields. Contrarily, those with electives in anesthesiology had higher rates of exposure to intubation (62% vs 18%, p < 0.05), IV insertion (69% vs 15%, p = 0.006), and bag-mask ventilation (62% vs 15%, p = 0.016) when compared to students who did not complete anesthesiology electives.

Motivation to learn skills

A total of >80% of students were highly motivated to learn skills (Table 4). Students were least motivated to learn how to perform IO insertions (77%) and DREs (74%). Of all factors tested, students interested in medical specialties were more motivated to learn IM injections (88% vs 76%, p = 0.039).

**Table 4:** Motivation for learning and likelihood of future use of an evidence-based list of procedural skills IV: intravenous; IO: intraosseous; IM: intramuscular; SC: subcutaneous; NG: nasogastric; IUD: intrauterine device; DRE: digital rectal examination

	Motivation		Likelihood	
Skill	Low/Very Low	High/Very High	Low/Very Low	High/Very High
Intubation	0.0%	94.3%	22.6%	39.6%
IV Insertion	0.0%	100%	9.4%	67.9%
Suturing	1.9%	92.5%	3.8%	86.8%
Phlebotomy	1.9%	96.2%	20.8%	41.5%
IO Insertion	3.8%	77.3%	18.9%	37.7%
IM Injections	1.9%	81.1%	1.9%	79.2%
SC Injections	3.8%	83.0%	3.8%	81.1%
Intradermal injections	1.9%	86.8%	3.8%	77.4%
NG Tube Insertion	0.0%	88.7%	22.6%	37.7%
Foley Catheter (Male)	1.9%	88.7%	39.6%	35.8%
Foley Catheter (Female)	1.9%	88.7%	35.8%	37.7%
IUD Insertion	3.8%	86.8%	32.1%	43.4%
DRE	7.5%	73.6%	20.8%	45.3%
Speculum Examination	3.8%	84.9%	18.9%	50.9%
Breast Examination	5.7%	81.1%	20.8%	45.3%
Bag-Mask Ventilation	0.0%	83.0%	28.3%	39.6%
Lumbar Puncture	0.0%	92.4%	24.5%	35.8%
Central Venous Catheter	0.0%	96.2%	22.6%	37.7%
Joint Aspiration	0.0%	88.7%	26.4%	35.8%
Superficial Wound Care	0.0%	94.3%	13.2%	60.3%
Throat Swabbing	0.0%	81.1%	5.7%	60.4%

Perceived likelihood of future skill use

Skills that participants felt they would use the most in their future practice included IV insertions (68%), ID injections (77%), IM injections (79%), SC injections (81%), and suturing (87%) (Table 4). The likelihood that students felt they would need to perform these skills in the future varied depending on demographic factors. For example, females felt a higher likelihood that they would need to perform IUD insertions (59% vs 14%, p < 0.001), DREs (59% vs 29%, p = 0.011), speculum examinations (66% vs 29%, p = 0.003) and breast examinations (63% vs 19%, *p* = 0.001) when compared to males. Additionally, students interested in rural centers felt more likely that they would need to perform throat swabs in the future (86% vs 57%, p = 0.022) compared to those that prefer to work in urban centers.

Career interest further impacted perceptions of likelihood for future skill performance. Surgically oriented students felt unlikely to need to perform IM injections (43% vs 92%, p < 0.001), SC injections (50% vs 92%, p = 0.001), ID injections (43% vs 85%, p = 0.012), and superficial wound care (43% vs 64%, p = 0.041). This contrasted with students possessing medical specialty interests, who reported a higher likelihood that they would perform IM injections (96% vs 66%%, p = 0.01), SC injections (100.0% vs 66%, p = 0.004), ID injections (92% vs 59%, p = 0.012) and breast examinations (63% vs 31%, p = 0.039) in the future. Students interested in a career in anesthesiology perceived a higher likelihood that they would need to perform IV insertions (100% vs 66%, p = 0.045), phlebotomy (100% vs 40%, p = 0.034), and CVC insertions (67% vs 36%, p = 0.026).

Correlation analysis: comfort from exposure to skills, and motivation to learn from the likelihood of using procedural skills

Spearman correlation analysis highlighted that there was a greater correlation between improved student comfort with increased exposure to skills such as: intubation (ρ = 0.818, p < 0.001) IV skills (ρ = 0.824, p < 0.001), phlebotomy (ρ = 0.818, p < 0.001), and SC injections (ρ = 0.78, p < 0.001). Relatively lower positive correlation coefficients were associated with skills, such as IUD insertion (ρ = 0.430, p = 0.001), speculum examination (ρ = 0.448, p = 0.001), and joint aspiration (ρ = 0.300, p = 0.029).

Correlation analysis between the motivation to learn a specific skill and the student’s perceived likelihood of using that skill in their future practice showed that students are more motivated to learn the skills of lumbar punctures (ρ = 0.407, p = 0.002), ID injections (ρ = 0.388, p = 0.004), and CVC insertion (ρ = 0.402, p = 0.003) if they envisioned using these skills in the future. There was a relatively decreased motivation to learn the skills of SC injections (ρ = 0.292, p = 0.034) and IV placement (ρ = 0.276, p = 0.026), despite students thinking they will use these skills in their practice.

## Discussion

This study investigated medical students' self-reported exposure, comfort in performance, motivation for learning, and the likelihood of future use of procedural skills at a Canadian university. While the majority of hands-on clinical training occurs later in medical education, some universities recognize the importance of integrating these skills earlier on. Despite efforts to integrate early skills experience into undergraduate medical training, there continues to be a disconnect between the expected standard of procedural skill competence and students’ self-reported confidence in their abilities [4-6].

Student comfort was strongly influenced by exposure as low comfort was reported for all skills except for suturing, IM, SC, IV, and ID injections, which received higher exposure ratings. This was supported by the correlation analysis, as increased exposure had a high correlation with increased comfort in these skills. Furthermore, students felt least comfortable performing Foley catheter insertion, lumbar puncture, CVC insertion, and joint aspiration, to which they received the least exposure. Low comfort in these latter three skills was expected due to their increased level of difficulty, while some skills may also be performed by higher-level learners or other healthcare team members during elective experiences. As clerkship students are expected to know how to perform these skills in some capacity, our results support early integration of these skills into pre-clerkship training to ameliorate comfort levels in clinical performance among medical students [4,5,9].

Students who completed surgical electives felt less comfortable with NG tube insertion and Foley Catheter insertion in males and females compared to those without previous surgical electives. These findings may be attributed to the performance of these tasks by nurses or other staff, or higher relevance to other specialties, which may preclude medical student learning. Contrarily, despite increased exposure, students were not consequently more comfortable in performing DREs. Motivation to learn DREs was the lowest of all skills, suggesting that while undesirable for students, practice is especially impactful in increasing comfort with performing DREs. To address these identified gaps in exposure and comfort among surgically oriented students, incorporating these skills into undergraduate surgical education programs or increasing exposure to these skills in undergraduate medical education may improve this lack of skills comfort.

Low exposure to skills such as NG tube insertion and intubations among students with medicine-based electives may be attributed to higher use of these skills in an operating room or emergency department setting [20,21]. In contrast, students who completed an elective in anesthesiology indicated increased exposure and comfort levels with the skills of intubation and IV insertion, further supporting that exposure to skills gained on clinical placements can significantly improve student comfort in performance. This underscores the importance of pre-clerkship electives in medical skill development [22].

Student motivation to learn skills was strongly associated with the perceived likelihood of future skills use. This was demonstrated as students interested in medical specialties felt more likely and were more motivated to perform IM injections. Similarly, suturing was a skill that students felt highly motivated to learn and for which they perceived a higher likelihood of use. Of all skills, students reported the highest motivation to learn IV insertions, intubations, phlebotomy, and superficial wound care; none of which were specifically associated with less exposure, the likelihood of future use, or comfort level in performance. This observation may be due to personal interest or general perceptions of skills in which all physicians should be competent [23].

The likelihood that students felt they would need to perform certain skills was influenced most notably by gender and career interest. Females felt more likely to perform IUD insertions, DREs, speculum examinations, and breast examinations compared to males, which may be accounted for by differences in career interests among genders [24-27]. Additionally, students with surgical career interests felt less likely that they would need to perform IM injections, SC injections, ID injections, and superficial wound care while students interested in medical specialties felt more likely to perform IM injections, SC injections, ID injections, and breast examinations in the future. This was further demonstrated as students interested in anesthesiology felt they had higher chances of performing IV insertions, phlebotomy, and CVC insertions in the future. To our knowledge, the relationship between gender, career interest, and the likelihood of future procedural skill use has yet to be reported but could be used to identify student groups that would benefit from more exposure to certain skills to improve student comfort and competence.

With knowledge of student exposure and comfort in procedural skills, undergraduate medical curriculum planners can account for differing pre-clerkship experiences as early exposure and repetition are paramount for medical students when learning procedural skills [28]. To determine which skills require optimization in their delivery, we show that baseline comfort and the effect of exposure on comfort with skill performance can be analyzed. Our results suggest that students gain the most benefit from skills such as intubation, IV insertion, and phlebotomy, which yielded high correlation coefficients with previous skill exposure. Furthermore, our study identified skills that benefit minimally by more exposure such as IUD insertion, speculum examination, and joint aspiration, allowing for undergraduate planning committees to optimize procedural skill learning approaches.

Although this study provides insight into pre-clerkship student experience with procedural skills, limitations exist. Participants were from one Canadian university although represented campuses located in separate provinces. Participants surveyed were applicants of the Pre-Clerkship Residency Exploration Program, which provides practical exposure to medical subspecialties and may account for the low interest in surgery among participants. The number of students interested in rural practice was also low. Additionally, while this study was quantitative, the use of qualitative methods may uncover factors that were not apparent here. Finally, all responses were based on self-assessment which may introduce bias. However, the literature suggests that self-perceptions of procedural skill competence is correlated with knowledge levels of clinical procedural skills and that self-perceived confidence translates to competence [29,30].

## Conclusions

This study supports that medical students report low comfort in performing procedural skills, despite high motivation to learn them. Comfort in skills performance was influenced by both career interest and elective experience. Students report higher comfort levels as exposure to these skills increases, and higher correlation coefficients suggest that undergraduate medical programs can particularly target intubation, IV insertion, and phlebotomy. Programs desiring to better prepare medical students for residency should integrate procedural skills with the largest gap in confidence such as Foley catheter insertion in males and females, lumbar puncture, and CVC insertion into curricula. Future research may benefit from designing qualitative methods to investigate factors that impact motivation for learning procedural skills while expanding studies to a regional or national level.
